# Predicting short-term outcomes in atrial-fibrillation-related stroke using machine learning

**DOI:** 10.3389/fneur.2023.1243700

**Published:** 2023-11-08

**Authors:** Eun-Tae Jeon, Seung Jin Jung, Tae Young Yeo, Woo-Keun Seo, Jin-Man Jung

**Affiliations:** ^1^Department of Neurology, Korea University Ansan Hospital, Korea University College of Medicine, Ansan, Republic of Korea; ^2^Department of Family Medicine, Gimpo Woori Hospital, Gimpo, Republic of Korea; ^3^Department of Neurology, Samsung Medical Center, Sungkyunkwan University School of Medicine, Seoul, Republic of Korea; ^4^Korea University Zebrafish Translational Medical Research Center, Ansan, Republic of Korea

**Keywords:** atrial fibrilation, machine learning, outcome, prediction model, ischemic stroke

## Abstract

**Background:**

Prognostic prediction and the identification of prognostic factors are critical during the early period of atrial-fibrillation (AF)-related strokes as AF is associated with poor outcomes in stroke patients.

**Methods:**

Two independent datasets, namely, the Korean Atrial Fibrillation Evaluation Registry in Ischemic Stroke Patients (K-ATTENTION) and the Korea University Stroke Registry (KUSR), were used for internal and external validation, respectively. These datasets include common variables such as demographic, laboratory, and imaging findings during early hospitalization. Outcomes were unfavorable functional status with modified Rankin scores of 3 or higher and mortality at 3 months. We developed two machine learning models, namely, a tree-based model and a multi-layer perceptron (MLP), along with a baseline logistic regression model. The area under the receiver operating characteristic curve (AUROC) was used as the outcome metric. The Shapley additive explanation (SHAP) method was used to evaluate the contributions of variables.

**Results:**

Machine learning models outperformed logistic regression in predicting both outcomes. For 3-month unfavorable outcomes, MLP exhibited significantly higher AUROC values of 0.890 and 0.859 in internal and external validation sets, respectively, than those of logistic regression. For 3-month mortality, both machine learning models exhibited significantly higher AUROC values than the logistic regression for internal validation but not for external validation. The most significant predictor for both outcomes was the initial National Institute of Health and Stroke Scale.

**Conclusion:**

The explainable machine learning model can reliably predict short-term outcomes and identify high-risk patients with AF-related strokes.

## 1. Introduction

Atrial fibrillation (AF) is a common cause of ischemic stroke, and AF-related strokes are associated with higher mortality and poorer functional outcomes than other ischemic stroke subtypes (1). The early identification of high-risk patients with poor functional outcomes is critical for maintaining a focus on available healthcare resources and improving outcomes in terms of prevention and early management. Accordingly, numerous studies have been conducted to predict vascular events, mortality, and functional outcomes in patients experiencing AF-related stroke events. Although originally used for thromboembolic risk assessment in AF outpatients, the CHADS_2_ score and its updated version, CHA_2_DS_2_VASc, can be used to effectively predict the prognosis of an AF-related stroke event (2–6). However, these models are simplified and do not incorporate clinical and laboratory parameters such as the severity of stroke symptoms at admission, serum inflammatory markers, and image-based features derived from the early stages of stroke. Furthermore, the clinical implications of these scores remain controversial in stroke patients (7–9), and it may be challenging to interpret features and values extracted from other studies for practical clinical applications (10).

Clinical risk scoring systems such as CHADS_2_, CHA_2_DS_2_-VASc, and ATRIA were originally developed for AF. However, their validation in stroke patients with AF, particularly in real-world settings with new oral anticoagulants (NOACs), has been rarely conducted. A nationwide multicenter study evaluated the scoring systems and presented unsatisfactory performance of the systems (9). This highlights the need for a new risk stratification approach tailored to secondary stroke prevention in AF patients.

Machine learning models offer various advantages over traditional parametric methods owing to their improved flexibility, capability to capture complex patterns, and good performance on large and high-dimensional datasets. All of these advantages are achieved without relying on strong assumptions, enabling machine learning to be utilized as a valuable tool in clinical practice. Therefore, it is desirable to develop novel prognostic methods that are easily interpretable and can improve risk stratification during the early stages of AF-related stroke.

Outcome prediction following ischemic stroke is generally performed using logistic regression as a statistical model using clinical and/or image-based features. However, the prediction results represent only the importance and linear directionality of the selected variables without any direct information regarding their priority. To overcome the limitations of conventional statistical and machine learning models, more accurate high-level machine learning techniques must be developed and applied (11). The machine-learning-based prediction of outcomes using information obtained during the early period after hospital arrival—including clinical, laboratory, and imaging findings—is a feasible method of formulating therapeutic plans and prognoses (11, 12). In this study, machine learning models were constructed and validated for the prediction of short-term outcomes in AF-related stroke patients based on various features acquired during early hospitalization using two independent multicenter prospective hospital-based registries.

## 2. Methods

### 2.1. Study population

**Figure 1:**
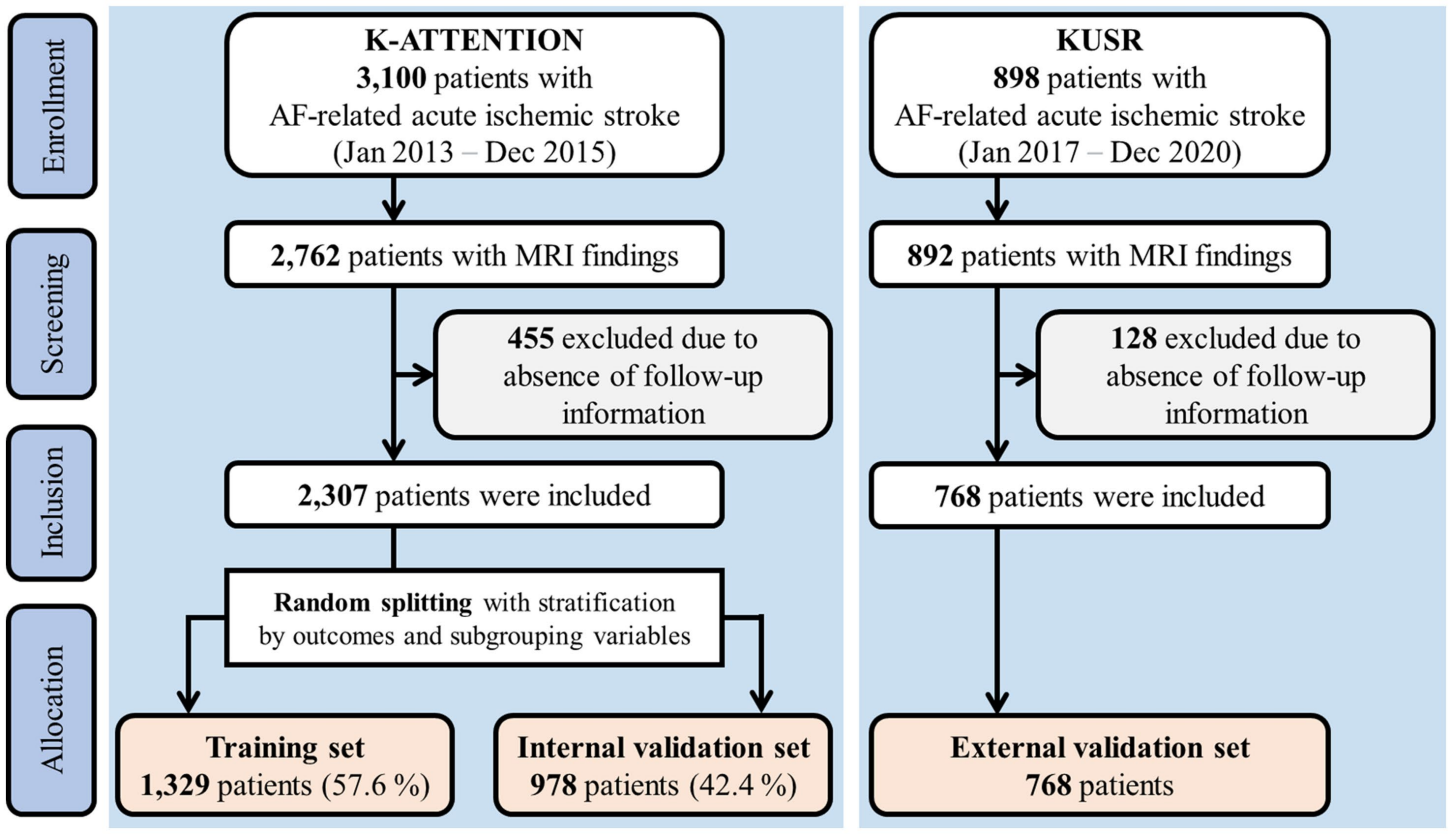
Flowchart of study subjects.

The internal and external validation sets used in this study are overviewed in Figure 1. One dataset was based on the Korean Atrial Fibrillation Evaluation Registry in Ischemic Stroke Patients (K-ATTENTION), which compiled medical information from AF-related stroke patients admitted within 7 days of symptom onset at 11 tertiary stroke centers in South Korea. K-ATTENTION has previously been used for model training, variable selection, and internal validation. The other dataset, used for external validation, comprised patients with AF-related stroke events extracted from the Korea University Stroke Registry (KUSR), collected from three Korean university hospitals (Anam, Ansan, and Guro branches). Common features among clinical, radiological, and laboratory findings acquired during early hospitalization were extracted from the two datasets to develop the models (11). Features with a loss of information pertaining to 3-month functional outcomes measured using the modified Rankin Scale (mRS) were excluded.

Approval was obtained from the institutional review boards of all participating centers. This study complied with the transparent reporting of a multivariable prediction model for individual prognosis or diagnosis reporting guidelines (13).

### 2.2. Definition of outcomes

The outcomes of interest were short-term outcomes following 90 days of the index stroke. Primary outcomes were defined as unfavorable functional outcomes with an mRS ≥ 3. The secondary outcome was mortality, defined as all-cause death within the 90-day period.

### 2.3. Data splitting and preprocessing

Approximately 40% of the patients from the K-ATTENTION registry were randomly selected and stratified by outcomes and variable subgroups (Figure 1). These data were used as the internal validation set, whereas the remaining data were allocated for training. Models were trained using a 10-fold cross-validation strategy.

Variables with a missing rate exceeding 20% were excluded, and missing values were imputed using multivariate imputation by chained equations (MICE) (14). Outliers were detected using an isolation forest (15) and replaced with the closest normal values from the training set. Supplementary Table S1 from the Supplementary Information lists all variables included in the analysis along with their missing rates.

### 2.4. Importance of variables and feature selection

The contribution of each variable to model prediction was evaluated using the Shapley additive explanations (SHAP) method (16). Positive and negative SHAP values indicate positive and negative effects, respectively, on the prediction score. A greedy backward selection method, namely, recursive feature elimination (17), was used to select the best feature set to maximize cross-validation performance by evaluating SHAP in every recursion. The mean absolute SHAP value for each variable was calculated as a ranking criterion representing the importance of each variable. The light gradient boosting machine (LightGBM) (18), a gradient-boosted tree-based model that can handle categorical variables, was used for SHAP evaluation.

The SHAP method was used to investigate the local interpretability of the developed LightGBM model. Model predictions were visualized for true-positive, true-negative, false-positive, and false-negative cases.

### 2.5. Model development

A logistic regression with L2 regularization was used as the baseline comparator. We tested two representative machine learning models, namely, LightGBM and the multi-layer perceptron (MLP). MLP is a feedforward neural network with fully connected layers. Hyperparameters were tuned using Bayesian optimization (19) to maximize the predictive performance of cross-validation. All details pertaining to hyperparameter settings are described in the Supplementary Methods and Supplementary Table S2. Models were calibrated using isotonic regression on the validation data during cross-validation. All methods used in this study were implemented in Python version 3.9.7 using Scikit-learn version 1.1.2.

### 2.6. Internal validation in different subgroup cohorts

The model performance of internal validation was evaluated in different subgroup cohorts. Age (≤64, 65–74, and ≥ 75 years), sex, hypertension, diabetes mellitus, type of AF (sustained and paroxysmal AF), and stroke recurrence (first-ever and recurrent stroke) were defined as subgroups.

### 2.7. Statistical analysis

Descriptive statistics were expressed as numbers (percentages), means (standard deviations), or medians (interquartile ranges). The Shapiro–Wilk test was used for normality, and Levene’s test was used for homoscedasticity. The chi-squared test, independent *t*-test, or Mann–Whitney *U*-test was used for comparison.

The area under the receiver operating characteristic curve (AUROC) was used as the primary outcome metric, as well as a guiding metric in all model training processes, including variable selection (recursive feature elimination) and hyperparameter tuning (Bayesian optimization). The AUROC was calculated and compared between models using the DeLong method (20). Furthermore, we evaluated the area under the precision–recall curve (AUPRC) of the models as an overall performance measure.

The detailed performance of each model was evaluated in terms of sensitivity, positive predictive value, and negative predictive value with net reclassification improvement (NRI) at low false positive rates (FPRs) of 5, 10, and 20%. A positive NRI value indicates superior reclassification performance by the new model compared to that of the reference model (21). We evaluated sensitivity at fixed FPR levels in the subgroups using the model that exhibited the best overall performance. Calibration errors were evaluated before and after calibration using the calibration curve and Brier score, which is the mean-squared error of predicted probability (22). We evaluated the model’s capability of risk stratification for the prediction of 3-month mortality. Four quartile strata were obtained from the prediction scores of the best-performing model, and survival curves were plotted using Kaplan–Meier estimation and compared using the log-rank test. The cutoff thresholds for investigating local interpretability were determined at an FPR level of 20%.

*p*-values were adjusted using the Benjamini–Hochberg method for multiple comparisons. The significance level was set at a *p*-value of <0.05.

## 3. Results

### 3.1. Baseline characteristics of study subjects

A total of 2,307 and 898 patients were included in the K-ATTENTION and KUSR groups, respectively (Figure 1). Table 1 presents a comparison between the two registries, including the general characteristics of the study participants, revealing an unfavorable functional outcome rate of 49.8% and the mortality rate of 11.6% 3 months following the index stroke. These results are compatible with unfavorable functional outcomes at 3 months poststroke (*p* > 0.05), whereas the models significantly differed in terms of 3-month mortality (*p* < 0.05). The patients in the two registries were comparable in terms of sex, initial National Institute of Health and Stroke Scale (NIHSS) score, hypertension, and history of vascular diseases, including coronary and peripheral artery diseases. However, patients in the KUSR group were generally older and had higher initial blood pressure and body mass index (BMI) values than those in the K-ATTENTION group. Furthermore, patients in the KUSR group were more likely to exhibit a severe pre-stroke functional status, persistent AF, and a history of congestive heart failure and diabetes mellitus, whereas they had a lower previous history of stroke. The initial imaging findings revealed significant differences in lesion lateralization, diffusion-weighted imaging (DWI) lesion patterns, and concomitant intracranial and extracranial artery stenosis.

**Table 1:** General characteristics of study participants.

Variable	All (*n* = 3,125)	K-ATTENTION (*n* = 2,307)	KUSR (n = 818)	*p* value^a^
*Demographics*				
Age, years	75.0 [67.0–80.0]	74.0 [67.0–80.0]	76.0 [67.0–82.0]	< 0.001^***^
Male sex, *n* (%)	1,628 (53.0%)	1,222 (53.0%)	406 (53.0%)	1.000
BMI, kg/m2	23.4 (3.4)	23.3 (3.4)	23.8 (3.5)	< 0.001^***^
*Initial clinical status*				
Initial DBP, mmHg	85.5 (15.7)	84.7 (14.9)	87.8 (17.6)	< 0.001^***^
Initial SBP, mmHg	145.5 (27.7)	144.3 (27.7)	148.9 (27.4)	< 0.001^***^
Initial pulse rate, beats per min	83.7 (21.4)	83.4 (21.4)	84.6 (21.4)	0.168
Initial NIHSS	7.0 [2.0–15.0]	7.0 [2.0–15.0]	6.0 [2.0–15.0]	0.412
*Pre-existing clinical status*				
Pre-stroke mRS	0 [0–1]	0 [0–1]	0 [0–3]	< 0.001^***^
CHADS_2_	2 [1–3]	2 [1–3]	2 [1–4]	< 0.001^***^
CHA_2_DS_2_VASc	3 [2–5]	3 [2–4]	4 [3–5]	< 0.001^***^
Stroke onset time, h	14.4 (18.0)	13.2 (6.3)	17.7 (33.8)	< 0.001^***^
*Pre-existing comorbidities*				
Stroke	916 (29.8%)	749 (32.5%)	167 (21.8%)	< 0.001^***^
Sustained AF	1,599 (52.0%)	1,174 (50.9%)	425 (55.5%)	0.030^*^
CHF	173 (5.6%)	95 (4.1%)	78 (10.2%)	< 0.001^***^
HTN	2,121 (69.0%)	1,587 (68.8%)	534 (69.7%)	0.665
DM	843 (27.4%)	603 (26.1%)	240 (31.3%)	0.006^**^
CAD	454 (14.8%)	332 (14.4%)	122 (15.9%)	0.327
PAD	41 (1.3%)	33 (1.4%)	8 (1.0%)	0.532
*Initial image findings*				
Lesion lateralization				< 0.001^***^
Rt. anterior	904 (29.4%)	817 (35.4%)	87 (11.4%)	
Lt. anterior	1,038 (33.8%)	798 (34.6%)	240 (31.3%)	
Posterior	670 (21.8%)	385 (16.7%)	285 (37.2%)	
Bilateral or diffuse multifocal	461 (15.0%)	307 (13.3%)	154 (20.1%)	
DWI lesion pattern				< 0.001^***^
Single corticosubcortical	669 (21.8%)	552 (23.9%)	117 (15.3%)	
Cortical	289 (9.4%)	201 (8.7%)	88 (11.5%)	
Subcortical (≥15 mm)	185 (6.0%)	152 (6.6%)	33 (4.3%)	
Subcortical (<15 mm)	190 (6.2%)	115 (5.0%)	75 (9.8%)	
Small scattered lesion in one vascular territory	330 (10.7%)	221 (9.6%)	109 (14.2%)	
Confluent and an additional lesion in one vascular territory	521 (17.0%)	405 (17.6%)	116 (15.1%)	
Multiple lesions in multiple vascular territories	646 (21.0%)	418 (18.1%)	228 (29.8%)	
Concomitant ICAS	1,466 (47.9%)	1,235 (53.5%)	231 (30.2%)	< 0.001^***^
Concomitant ECAS	611 (20.1%)	548 (23.8%)	63 (8.2%)	< 0.001^***^
*Laboratory findings*				
WBC, 10^3^/μL	8.3 (3.2)	8.3 (3.1)	8.3 (3.4)	0.903
Hb, g/dL	13.5 (2.0)	13.5 (2.0)	13.5 (2.0)	0.876
PLT, 10^3^/mcL	205.0 (72.9)	205.0 (76.5)	204.8 (60.9)	0.929
hs-CRP, mg/L	4.3 (16.0)	2.8 (11.3)	8.6 (24.6)	< 0.001^***^
Initial glucose, mg/dL	142.8 (72.5)	141.2 (76.6)	147.3 (58.8)	0.047^*^
Fasting glucose, mg/dL	122.4 (43.9)	121.9 (43.6)	123.7 (44.7)	0.335
HbA1c, %	6.1 (2.5)	6.1 (2.1)	6.3 (3.2)	0.018^*^
Total cholesterol, mg/dL	162.0 (38.9)	162.8 (37.8)	159.7 (41.6)	0.071
TG, mg/dL	100.0 (65.2)	97.6 (61.8)	106.5 (73.4)	0.001^**^
HDL, mg/dL	46.6 (14.5)	46.9 (14.9)	46.0 (13.3)	0.148
LDL, mg/dL	99.4 (33.6)	100.7 (33.2)	95.8 (34.7)	< 0.001^***^
AST, U/L	26.0 [21.0–33.0]	26.0 [21.0–33.0]	26.0 [21.0–33.0]	0.908
ALT, U/L	19.0 [14.0–27.0]	18.0 [14.0–26.0]	19.0 [14.0–28.0]	0.546
ALP, IU/L	93.4 (65.1)	99.0 (73.0)	79.6 (35.2)	< 0.001^***^
Total bilirubin, mg/dL	0.9 (0.5)	0.9 (0.5)	0.8 (0.5)	< 0.001^***^
Uric acid, mg/dL	5.4 (2.6)	5.5 (2.8)	5.1 (1.7)	0.003^**^
Serum creatinine, mg/dL	0.9 [0.7–1.1]	0.9 [0.7–1.1]	0.9 [0.8–1.2]	< 0.001^***^
CrCl, mL/min	64.8 [46.2–85.4]	61.4 [42.7–81.2]	75.5 [57.4–91.2]	< 0.001^***^
Fibrinogen, mg/dL	321.3 (120.9)	316.9 (125.0)	337.9 (102.9)	< 0.001^***^
Homocysteine, μmol/L	12.7 (36.8)	13.2 (43.4)	11.6 (6.0)	0.348
CK-MB, ng/mL	3.6 (6.4)	3.5 (6.4)	3.9 (6.4)	0.163
FFA, μEq/L	866.1 (467.7)	894.2 (557.1)	848.6 (401.2)	0.129
*Thrombolytic treatment*				
Recanalization therapy				< 0.001^***^
None	2,162 (70.4%)	1,641 (71.1%)	521 (68.0%)	
IV	505 (16.4%)	393 (17.0%)	112 (14.6%)	
IA	195 (6.3%)	133 (5.8%)	62 (8.1%)	
IV + IA	201 (6.5%)	140 (6.1%)	61 (8.0%)	
*3-month outcomes*				
Mortality	355 (11.6%)	294 (12.7%)	61 (8.0%)	< 0.001^***^
Unfavorable outcomes	1,530 (49.8%)	1,136 (49.2%)	394 (51.4%)	0.312

a *p*-value of the chi-squared test, independent *t*-test, or Mann–Whitney U test for comparing the difference between the internal validation set (K-ATTENTION) and the external validation set (KUSR). ^*^p < 0.05, ^**^p < 0.01, ^***^p < 0.001.

Values are represented as *n* (%), mean (SD), or median [95% CIs]. BMI, body mass index; DBP, diastolic blood pressure; SBP, systolic blood pressure; NIHSS, National Institute of Health and Stroke Scale; mRS, modified Rankin Scale; AF, atrial fibrillation; CHF, congestive heart failure; DM, diabetes mellitus; CAD, coronary artery disease; PAD, peripheral artery disease; DWI, diffusion-weighted image; ICAS, intracranial artery stenosis; ECAS, extracranial artery stenosis; WBC, white blood cell; PLT, platelet; TG, triglyceride; HDL, high-density lipoprotein; LDL, low-density lipoprotein; CrCl, creatinine clearance; FFA, free fatty acid; IV, intravenous; IA, intra-arterial.

### 3.2. Model performance

**Figure 2:**
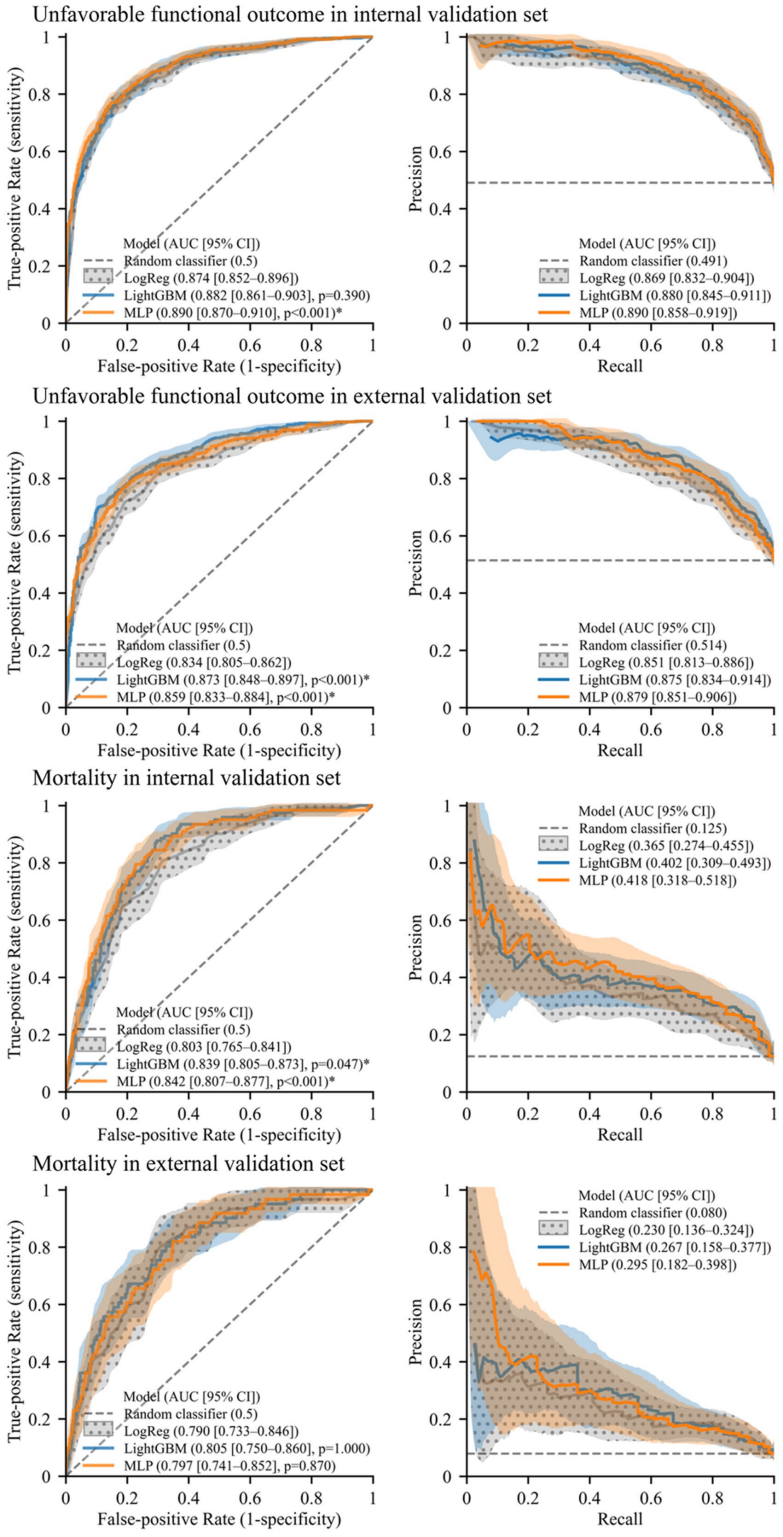
Receiver operating characteristics curves and precision–recall curves illustrating model performance. Solid lines and shades represent mean curves and 95% confidence interval areas, respectively. For the baseline model (logistic regression, “LogReg”), confidence intervals are represented with a polka dot pattern. An asterisk (*) indicates significantly higher AUROC than the baseline model (*p* < 0.05, Benjamini–Hochberg corrected).

An evaluation of model performance is presented in Figure 2, indicating that the machine learning models outperform logistic regression in predicting both outcomes. In the prediction of unfavorable functional outcomes, MLP obtained an AUROC value of 0.890 on the internal validation set, representing a significant improvement over the AUROC value of 0.874 obtained by logistic regression. In the external validation set, both LightGBM (0.873) and MLP (0.859) achieved significantly higher AUROC values than those of logistic regression (0.834).

In the prediction of mortality, both LightGBM (0.839) and MLP (0.842) attained significantly higher AUROC values than logistic regression (0.803) on the internal validation set. On the external validation set, the AUROC values of LightGBM (0.805) and MLP (0.797) were also higher than that of logistic regression (0.790) although not significantly so. Furthermore, the machine learning models exhibited higher AUPRC values than those of logistic regression for predicting both outcomes in each validation set.

The cross-validation performance and calibration curves with the Brier scores of the models are presented in Supplementary Table S3 and Supplementary Figure S1, respectively. MLP was used to obtain four quartile strata, and mortality risk stratification was evaluated with pairwise comparisons between the survival curves of the strata using the log-rank test (Supplementary Figure S2). For the internal validation set, all *p*-values for pairwise comparisons were less than 0.0001, except for those between the first and second quartiles. For the external validation set, *p*-values for the pairwise comparisons were 0.0002 or less, except for those between adjacent quartiles.

### 3.3. Performance comparison according to subgroup

Model performance in predicting unfavorable functional outcomes was consistent across all subgroups, with the lowest AUROC and AUPRC values in patients with recurrent stroke and those aged <65 years, respectively. Both machine learning models exhibited comparable or superior performance to that of logistic regression, with MLP significantly outperforming logistic regression in most subgroups (*p* < 0.05) except for patients aged <65 and > 74 years and those with recurrent strokes. No significant differences were observed between the two machine learning models or between logistic regression and LightGBM.

Supplementary Figure S3 shows the results of a performance comparison in the prediction of 3-month mortality across subgroups. Performance was also consistent across subgroups, with machine learning models exhibiting superior performance for all subgroups except patients aged <65 years.

**Figure 3:**
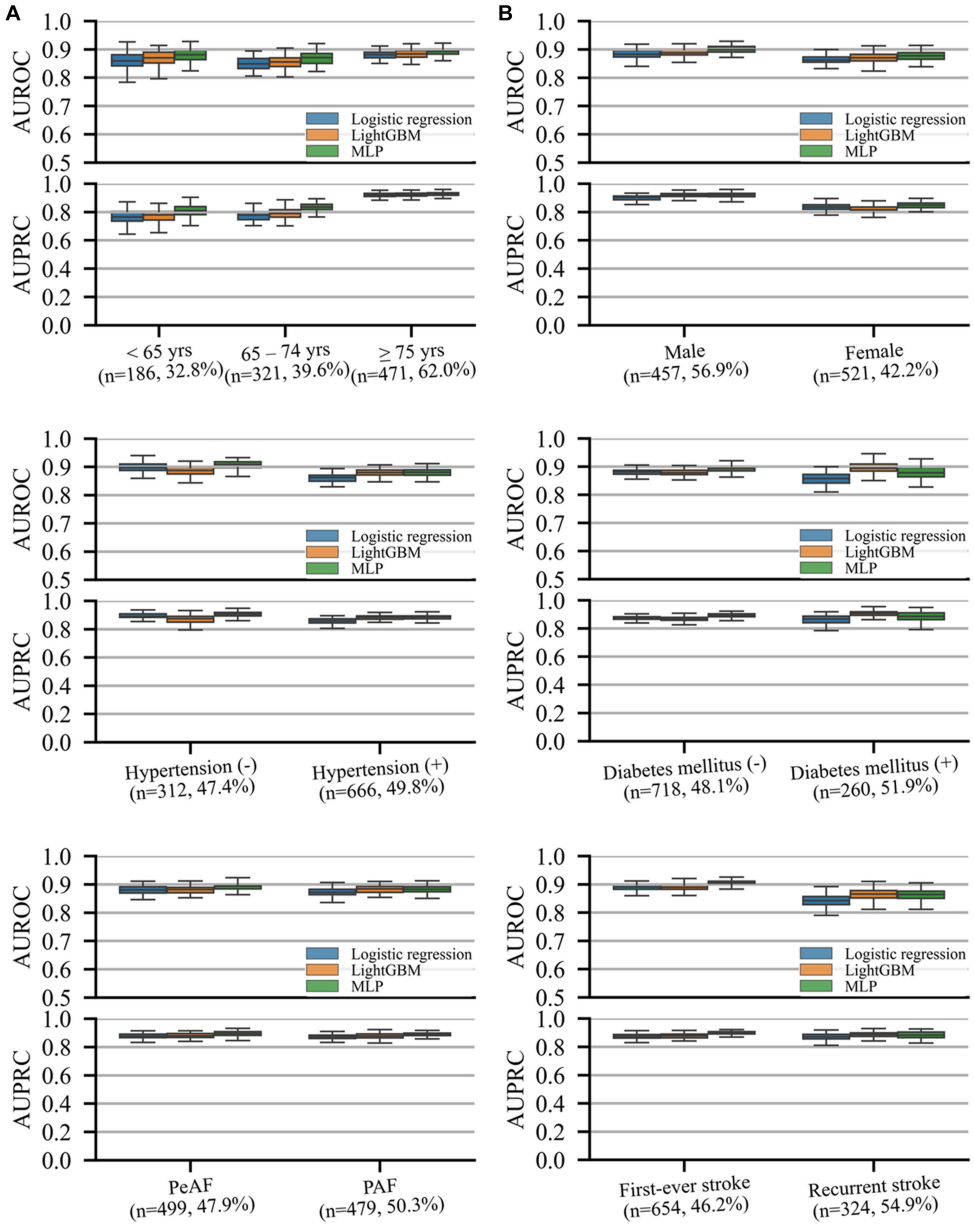
AUROC and precision–recall curves representing performance on different subgroup cohorts for the prediction of unfavorable functional outcomes. The (*n* = A, B%) notation for each subgroup indicates the number of samples in the test set **(A)** and prevalence rate of the outcome **(B)** of the subgroup. Box plots are plotted with whiskers of 1.5 times the interquartile ranges. AUPRC, area under the precision–recall curve; AUROC, area under the receiver operating characteristics curve; DM, diabetic mellitus; HTN, hypertension; PAF, paroxysmal atrial fibrillation (AF); PeAF, persistent atrial fibrillation (AF).

Low-FPR sensitivity results in the subgroup cohorts for predicting unfavorable functional outcomes and mortality are presented in Supplementary Figures S4, S5, respectively. Detailed performance and NRI at low FPRs for the prediction of unfavorable functional outcomes and mortality are presented in Supplementary Tables S4, S5, respectively (see Figure 3).

### 3.4. Importance of variables for prediction of unfavorable outcomes

**Figure 4:**
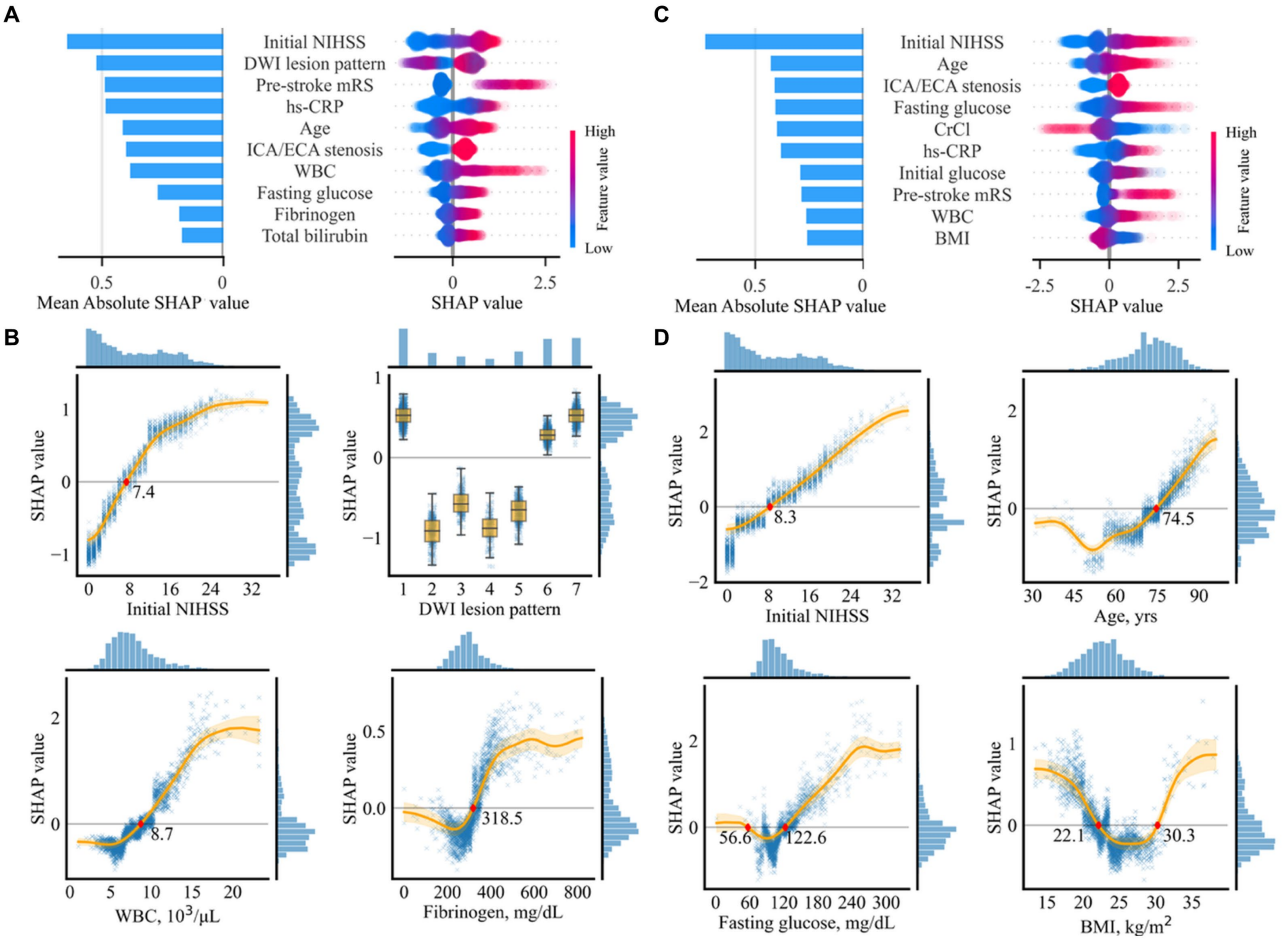
Importance of selected variables for prediction of unfavorable functional outcomes **(A,B)** and mortality **(C,D)**. **(A,C)** Individual influences of every value and overall contributions to the model prediction of the top 10 variables are represented as a dot on the right and bar on the left, respectively. In the plot on the right, red dots indicate high values in continuous/ordinal variables. Positive and negative SHA*p* values indicate positive contributions resulting in higher prediction scores and negative contributions resulting in lower prediction scores, respectively. **(B,D)** Partial SHAP dependence plots for four representative variables. Histograms on the right and the top axes of each plot indicate SHAP distributions and variable values, respectively. The original labels of the numeric codes are as follows: 1, single corticosubcortical; 2, cortical; 3, subcortical (≥ 15 mm); 4, subcortical (< 15 mm); 5, small scattered lesion in one vascular territory; 6, confluent and an additional lesion in one vascular territory; and 7, multiple lesions in multiple vascular territories. BMI, body mass index; CrCl, creatinine clearance; NIHSS, National Institute of Health Stroke Scale; DWI, diffusion-weighted imaging; WBC, white blood cell; hs-CRP, highly sensitive C-reactive protein; ECA, extracranial artery; ICA, intracranial artery.

Out of the 43 variables, 34 were selected in the variable selection process, with the 10 most important variables summarized in Figure 4A. The most important variables were the initial NIHSS score, followed by DWI lesion pattern, pre-stroke mRS, and hs-CRP.

Partial SHAP dependence plots for the four representative variables among the top 10 are displayed in Figure 4B, with those for the other six variables presented in Supplementary Figure S6. Thus, ≥ 7.4 points in the initial NIHSS score, having a specific pattern of lesion, including single corticosubcortical lesion, confluent and an additional lesion in one vascular territory, or multiple lesions in multiple vascular territories, ≥ 8,700 cells/μL in white blood cell (WBC) count, ≥ 318.3 mg/dL in fibrinogen, ≤ 22.5 kg/m^2^ in BMI, ≥ 3 in pre-stroke mRS, bilateral or diffuse multifocal lesion lateralization, ≤ 12.9 g/dL in hemoglobin, ≥ 74.3 years in age, and ≤ 51.8 mL/min in creatine clearance contributed to a higher risk of the unfavorable functional outcome. The SHAP values associated with the 10 most important variables exhibited a pattern comparable to linear association. WBC revealed a sigmoid pattern, fibrinogen displayed a J-shaped pattern, and other variables revealed complex linear, sigmoid, and J-shaped patterns.

Supplementary Figure S7 presents the local interpretability of LightGBM for the prediction of unfavorable outcomes with individual cases on an external validation set.

### 3.5. Importance of variables for prediction of mortality

Out of the 16 selected variables, the 10 most important are summarized in Figure 4C. The most important variable was the initial NIHSS score, followed by age, concomitant intracranial/extracranial steno-occlusion, fasting glucose, and creatinine clearance.

Partial SHAP dependence plots for four representative variables are displayed in Figure 4D, with those for the other six variables presented in Supplementary Figure S8. In summary, ≥ 8.2 in the initial NIHSS score, ≥ 74.5 years in age, ≤ 56.6 mg/dL or ≥ 122.6 mg/dL in fasting glucose, and ≤ 22.1 or ≥ 30.3 kg/m^2^ in BMI, ≥ 4 in pre-stroke mRS, ≥ 87.2 IU/L in ALP, ≤ 4.0 mg/dL, or ≥ 7.0 mg/dL in uric acid, multiple lesions in multiple vascular territories, presence of concomitant intracranial or extracranial stenosis, and ≥ 8,300 cells/μl in WBC count predicted mortality. Most association patterns between SHAP values and variables were near-linear, sigmoid, J-shaped, or combinations of the three. BMI and uric acid levels revealed U-shaped patterns.

The local interpretability of LightGBM for the prediction of mortality is demonstrated in Supplementary Figure S9 by presenting individual cases through model prediction on the external validation set.

## 4. Discussion

We trained MLP and LightGBM machine learning models to predict unfavorable outcomes and mortality in AF-related stroke patients over a 3-month period using two separate datasets. All models were validated internally and externally, with the machine learning models exhibiting higher predictive power than logistic regression for both outcomes. Similar trends were consistently observed across pre-specified subgroups, including age, sex, hypertension, diabetes mellitus, type of AF, and stroke recurrence. Overall, the machine learning models reliably predicted unfavorable outcomes and mortality in AF-related stroke patients. We identified influential variables through SHAP values to improve model explainability and identify high-risk patients with poor outcomes.

The initial NIHSS score, which reflects initial stroke severity, was the most influential variable with the highest SHAP value in determining short-term prognoses (unfavorable outcomes and mortality) following AF-related stroke. This finding is consistent with those of previous studies, which demonstrated an association between the initial NIHSS score and poor outcomes (10, 23) and mortality (8, 24) after the occurrence of stroke. Similarly, we observed linear associations between the initial NIHSS score and both poor functional outcomes and mortality, with cutoff values of 7.4 and 8.2, respectively. Thus, the risk of mortality increased linearly with an initial NIHSS score exceeding 8.2.

Patient age was the second most significant variable affecting mortality, with a J-shaped association pattern. Patients aged 74.5 years and older exhibited a higher risk of mortality, which increased linearly with age. However, the magnitude of negative association decreased in patients aged 52 years and younger. The DWI lesion pattern was the second most influential variable for unfavorable functional outcomes. AF-related stroke patients exhibited a higher risk of poor functional outcomes when they had infarct patterns with single cortico-subcortical lesions, confluent and additional lesions in one vascular territory, or multiple lesions in different vascular territories. The size and number of ischemic lesions may indicate the burden of embolus, suggesting an association with functional outcomes.

Concomitant vascular diseases are frequently observed in AF patients because they share several risk factors and pathophysiological features with atherosclerosis (25, 26). Concomitant carotid atherosclerosis was identified as an important risk factor for short-term outcomes in this study. This result is consistent with previous results, demonstrating that carotid atherosclerosis predicts recurrent vascular events and mortality in AF-related stroke patients (26). However, this study is the first to determine an association with poor functional outcomes. The pre-stroke mRS score, representing the degree of functional disability prior to the index stroke, is a well-known robust predictor of prognosis following stroke (27). We observed that a pre-stroke mRS of 3 or higher is associated with poor short-term functional outcomes, whereas the association with mortality increased almost linearly for each single-point increase in pre-stroke mRS of 4 or higher.

WBC and fibrinogen levels exhibited sigmoid patterns according to SHAP values. The leukocyte count, a marker of inflammatory response, is associated with short-and long-term clinical outcomes following acute strokes (28, 29). Fibrinogens play crucial roles in the coagulation cascade and inflammation. Increased fibrinogen levels are associated with functional outcomes (30, 31). Notably, the cutoff values for poor prognosis in this study were similar to those discovered previously. The association between fasting glucose levels revealed a J-shaped sigmoidal pattern. An association with lower mortality rates was also observed with glucose levels ranging from 56.6 to 122.6 mg/dL. The lower and upper cutoff values were comparable to the blood glucose levels for hypoglycemia and diagnostic criteria for diabetes mellitus, respectively. Hyperglycemia may contribute to poor outcomes in stroke patients through several mechanisms, including an increased risk of cerebral edema (32, 33), impaired blood flow regulation (34), and increased oxidative stress (35). Hypoglycemia may also result in poor outcomes owing to impaired brain function as the brain requires a constant supply of glucose. Additionally, hypoglycemia can induce ischemic stroke by increasing the levels of inflammatory markers, platelet activation, and fibrinogen formation (36, 37).

Machine learning may provide significant advantages in medical practice by uncovering complex non-linear patterns within medical data and enhancing predictive accuracy vital for optimizing patient care. Machine learning models are also adept at tailoring predictions to individual patient profiles, aligning with the principles of personalized medicine. Moreover, they serve as powerful clinical decision support tools, providing data-driven insights that enhance the decision-making capabilities of healthcare practitioners, ultimately improving patient outcomes.

The machine learning models achieved robustness through internal and external validation sets based on two separate datasets with distinct patient characteristics. However, this study had some limitations. First, our models may have been overfitted to the Korean population, which would present a challenge in generalizability. The enrolled population was Asian, and most patients were treated under the Korean medical system covered by national health insurance, which improved the accessibility of medical services and standardization of treatment processes. Asian populations have been associated with a higher bleeding tendency than thrombotic risk compared to Western populations (38). Ethnic differences in fibrinogen levels, one of the 10 highest SHAP variables, have also been reported (39, 40). However, these national and ethnic differences cannot be considered by machine learning models. Second, some variables associated with outcomes in AF-related stroke (41, 42), including D-dimer and N-terminal pro-B-type natriuretic peptides, were not considered, owing to an excessive number of missing values. To improve model interpretability, we used categorized ischemic lesion patterns in place of raw brain magnetic scans. However, this approach may limit the utilization of potential information embedded in magnetic resonance images. Finally, although the machine learning models exhibited higher predictive power than logistic regression, a comparison in terms of AUROC and AUPRC did not reveal marked differences and these differences were statistically significant. Small differences were observed in terms of sensitivity at low FPR levels as each of the three models assumed the same number of variables. As displayed in Figure 4, most of the association patterns revealed roughly linear contributions. Generally, linear regression is a simple and powerful tool for modeling linear relations, whereas machine learning models can capture complex non-linear relationships between variables. Consequently, if the relationship between variables is primarily linear, the additional complexity of a machine learning model might not be conducive in terms of predictive accuracy. To improve model performance, artificial data generation techniques, such as synthetic minority oversampling, may be useful (43). The prevalence of outcomes for future model applications was not artificially increased, instead allowing the class imbalance to remain unmodified. To ensure that comparable performance can be expected when the models are applied to other independent datasets, we handled outliers using a popular automated method. Furthermore, we verified model performance in terms of AUPRC, with a more accurate reflection of practical performance than the AUROC (44).

Machine learning models can be used to predict short-term outcomes, including unfavorable outcomes and mortality over a 3-month period, and identify high-risk patients with poor outcomes in AF-related stroke. The initial NIHSS score was the most important factor influencing short-term prognosis. Because our results are restricted to Korean stroke patients, further validation is necessary to ensure that the models and selected features can be applied to all AF-related stroke patients.

## Data availability statement

The raw data supporting the conclusions of this article will be made available by the authors, without undue reservation.

## Ethics statement

The studies involving humans were approved by Korea University Ansan Hospital IRB. The studies were conducted in accordance with the local legislation and institutional requirements. The ethics committee/institutional review board waived the requirement of written informed consent for participation from the participants or the participants' legal guardians/next of kin because this is a retrospective study.

## Author contributions

W-KS and J-MJ designed the study and collected the data. E-TJ completed the machine learning and statistical analysis. E-TJ and SJ wrote the manuscript. J-MJ is responsible for the overall content as the guarantor. All authors contributed to the article and approved the submitted version.
