# Supplementary material for: Predicting short-term outcomes in atrial-fibrillation-related stroke using machine learning
Source: Front Neurol. 2023 Nov 8;14:1243700. doi: 10.3389/fneur.2023.1243700 (PMC10663332; doi:10.3389/fneur.2023.1243700)
Supplement: Supplementary file 1 [file Data_Sheet_1.docx]

**Supplementary materials**

**Predicting short-term outcomes in atrial-fibrillation-related stroke using machine learning**

**Supplementary Methods**

**Supplementary Tables**

Table S1. Missing rate and variable type of included variables

Table S2. Hyperparameter tuning results

Table S3. Cross-validation performance

Table S4. Detailed performance and net reclassification improvement at low false positive rates for predicting 3-month unfavorable functional outcomes

Table S5. Detailed performance and net reclassification improvement at low false positive rates for prediction of 3-month mortality

**Supplementary Figures**

Figure S1. Calibration curves before and after calibration of the models

Figure S2. Survival curves for the four quartile strata obtained from the model for predicting 3-month mortality.

Figure S3. Performance on different subgroup cohorts for prediction of 3-month mortality

Figure S4. Sensitivity at low false positive rates in different subgroup cohorts for the prediction of 3-month unfavorable functional outcomes

Figure S5. Sensitivity at low false positive rates in different subgroup cohorts for the prediction of 3-month mortality

Figure S6. Partial SHAP dependence plots for six representative variables for the prediction of 3-month unfavorable functional outcomes

Figure S7. Local interpretability of the developed gradient-boosted tree-based model for predicting 3-month unfavorable functional outcomes

Figure S8. Partial SHAP dependence plots for six representative variables for the prediction of 3-month mortality

Figure S9. Local interpretability of the developed gradient-boosted tree-based model for predicting 3-month mortality.

**Supplementary Methods**

Models

We tested the following three models for the outcome prediction.

- Logistic regression. We used the logistic regression class from the Scikit-learn library (version 1.1.2). Among the arguments, the solver and penalty were set as “lbfgs” and “l2,” respectively. The remaining arguments remained as default settings, except for C, which is an inverse of the regularization strength. The value of argument C was selected using Bayesian optimization by maximizing the area under the receiver operating characteristic (ROC) curve of the validation set.
- LightGBM. We used the class LGBMClassifier from the LightGBM library (version 3.3.2).^1^ Among the arguments, num_leaves (maximum tree leaves), n_estimators (number of boosted trees), min_split_gain (minimum loss reduction required to make a further partition on a leaf node of the tree), min_child_weight (minimum sum of instance weight needed in a leaf), min_child_samples (minimum number of data needed in a leaf), and reg_lambda (L2 regularization term on weights) were tuned using Bayesian optimization by maximizing AUROC on the validation set. Model training was performed when no improvement was observed in the AUROC of the validation set over ten consecutive training iterations. The remaining arguments were maintained at the default settings.
- Multi-layer perceptron. We used the sequential and class layers in the Keras module from the TensorFlow-GPU library (version 2.4.0). The base architecture consists of an input layer, a hidden layer with 64 nodes, a batch normalization layer, a dropout layer, and an output layer. Nadam (Adam optimizer with the Nesterov technique) was used as the optimizer, and the learning rate was set to 0.005. The activation function was an exponential linear unit (ELU). We determined the additional depth following the existing hidden layer, argument momentum of the batch normalization function, rate of the drop-out layer, and l of the l2 function in the hidden layers using Bayesian optimization by maximizing the AUROC on the validation set. The additional depth is the number of sets, and each set consists of a dense layer with 32 nodes and a batch normalization layer. Model training was performed when no improvement was observed in the AUROC of the validation set over 20 consecutive training iterations. The remaining arguments were maintained at default settings.

The search spaces and selected values for the hyperparameters were represented in Table S2.

^1^Guolin Ke, Qi Meng, Thomas Finley, Taifeng Wang, Wei Chen, Weidong Ma, Qiwei Ye, Tie-Yan Liu. "LightGBM: A Highly Efficient Gradient Boosting Decision Tree". Advances in Neural Information Processing Systems 30 (NIPS 2017), pp. 3149-3157.

**Supplementary Tables**

| **Table S1. Missing rate and variable type of included variables** | | | |
| --- | --- | --- | --- |
| **Variables** | **Variable type** | **Missing rate, %** | |
|  |  | **K-ATTENTION** | **KUSR** |
| **Demographics** |  |  |  |
| Age | Continuous | 0 | 0 |
| Sex | Categorical | 0 | 0 |
| BMI | Continuous | 4.9 | 2.9 |
| Smoking* | Categorical | 0 | 0 |
| **Initial clinical status** |  |  |  |
| Initial DBP | Continuous | 0 | 0.1 |
| Initial SBP | Continuous | 0 | 0.1 |
| Initial pulse rate | Continuous | 0 | 0.3 |
| Initial NIHSS | Continuous | 0.5 | 0 |
| **Pre-existing clinical status** |  |  |  |
| Pre-stroke mRS | Continuous | 0 | 7 |
| Stroke onset time | Continuous | 2.3 | 0.3 |
| **Pre-existing comorbidity** |  |  |  |
| Previous history of stroke | Categorical | 0 | 0 |
| Previous history of TIA | Categorical | 0 | 0 |
| AF type | Categorical | 0 | 0 |
| CHF | Categorical | 0 | 0 |
| HTN | Categorical | 0 | 0 |
| DM | Categorical | 0 | 0 |
| CAD | Categorical | 0 | 0 |
| PAD | Categorical | 0 | 0 |
| **Initial image findings** |  |  |  |
| Lesion lateralization* | Categorical | 0 | 0 |
| DWI lesion pattern* | Categorical | 10.4 | 0 |
| ICA/ECA stenosis | Categorical | 0 | 0 |
| **Laboratory findings** |  |  |  |
| WBC | Continuous | 0.5 | 0.1 |
| Hb | Continuous | 0.6 | 0.1 |
| PLT | Continuous | 0.8 | 0.1 |
| hs-CRP | Continuous | 8.4 | 3.1 |
| Initial glucose | Continuous | 6.1 | 2.7 |
| HbA1c | Continuous | 15.4 | 6.9 |
| Total cholesterol | Continuous | 1.8 | 1.2 |
| TG | Continuous | 10.7 | 1.8 |
| HDL | Continuous | 10.3 | 1.8 |
| LDL | Continuous | 9.2 | 2.2 |

| **Table S1. (*Continued.*)** | | | |
| --- | --- | --- | --- |
| **Variables** | **Variable type** | **Missing rate, %** | |
|  |  | **K-ATTENTION** | **KUSR** |
| AST | Continuous | 2.1 | 0 |
| ALT | Continuous | 2.1 | 0 |
| ALP | Continuous | 19.2 | 2 |
| Total bilirubin | Continuous | 13.4 | 0.3 |
| Uric acid | Continuous | 18.2 | 3.9 |
| Serum creatinine | Continuous | 1.9 | 0 |
| CrCl | Continuous | 0 | 0 |
| Fibrinogen | Continuous | 19.8 | 35.1 |
| aPTT | Continuous | 11.4 | 0.1 |
| PT | Continuous | 2.6 | 1.3 |
| Fasting glucose | Continuous | 11.8 | 3.1 |
| **Thrombolytic treatment** |  |  |  |
| Recanalization therapy* | Categorical | 0 | 1.3 |
| * implies categorical variables having more than three categories. Smoking status was grouped into nonsmoker, ex-smoker (<5 years), ex-smoker (≥5 years), and current smoker. Lesion lateralization was grouped into right anterior, left anterior, posterior, and bilateral or diffuse multifocal ischemic lesion. DWI lesion pattern grouped into single corticosubcortical lesion, cortical lesion, subcortical lesion (< 15 mm), subcortical lesion (≥ 15 mm), small scattered lesion in one vascular territory, confluent and an additional lesion in one vascular territory, and multiple lesions in multiple vascular territories. Recanalization therapy grouped into none versus intravenous (IV) versus intra-arterial (IA) versus IV + IA.  Abbreviations: BMI, body mass index; SBP, systolic blood pressure; DBP, diastolic blood pressure; NIHSS, National Institute of Health Stroke Scale; mRS, modified Rankin Scale; AF, atrial fibrillation; CHF, congestive heart failure; HT, hypertension; DM, diabetes mellitus; CAD, coronary artery disease; PAD, peripheral artery disease; DWI, diffusion-weighted image; ICAS, intracranial artery stenosis; ECAS, extracranial artery stenosis; WBC, white blood cell; CRP, C-reactive protein; TG, triglyceride; HDL, high-density lipoprotein; LDL, low-density lipoprotein; CrCL, creatinine clearance. | | | |

| **Table S2. Hyperparameter tuning results** | | | | | |
| --- | --- | --- | --- | --- | --- |
|  | **Model** | **Hyperparameter** | **Type** | **Search space (min, max)** | **Selected value** |
| **3-month unfavorable functional outcome** | | | | | |
|  | Logistic regression | C | float | (0.1, 10) | 8.5779 |
|  | LightGBM | num_leaves | integer | (40, 130) | 69 |
|  |  | n_estimators | integer | (70, 300) | 86 |
|  |  | min_split_gain | float | (0.01, 0.2) | 0.1636 |
|  |  | min_child_weight | float | (0, 0.01) | 0.0089 |
|  |  | min_child_samples | integer | (5, 100) | 51 |
|  |  | reg_lambda | float | (0, 0.3) | 0.3537 |
|  | Multi-layer perceptron | additional depth† | integer | (0, 3) | 1 |
|  |  | BN momentum | float | (0.8, 0.99) | 0.8178 |
|  |  | drop-out rate | float | (0.6, 0.7) | 0.6097 |
|  |  | l2 regularization strength | float | (0, 0.4) | 0.1246 |
| **3-month mortality** | | | | | |
|  | Logistic regression | C | float | (0.1, 10) | 8.7311 |
|  | LightGBM | num_leaves | integer | (40, 130) | 115 |
|  |  | n_estimators | integer | (70, 300) | 293 |
|  |  | min_split_gain | float | (0.01, 0.2) | 0.0758 |
|  |  | min_child_weight | float | (0, 0.01) | 0.0059 |
|  |  | min_child_samples | integer | (5, 100) | 39 |
|  |  | reg_lambda | float | (0, 0.3) | 0.0543 |
|  | Multi-layer perceptron | additional depth† | integer | (0, 3) | 2 |
|  |  | BN momentum | float | (0.8, 0.99) | 0.9839 |
|  |  | drop-out rate | float | (0.6, 0.7) | 0.6190 |
|  |  | l2 regularization strength | float | (0, 0.4) | 0.2826 |
| †This denotes the number of additional sets and each set consisted of a dense layer with 32 nodes and a batch normalization layer. The additional sets positioned between the existing hidden layer and the drop-out layer. Abbreviation: BN, batch normalization. | | | | | |

| **Table 3.** **Cross-validation performance** | | | | | | | |
| --- | --- | --- | --- | --- | --- | --- | --- |
| **Model** | **AUROC** | **AUPRC** | **Brier score** | **ACC, %** | **Precision** | **Recall** | **F1 score** |
| **3-month unfavorable functional outcome** | | | | | | | |
| LogReg | 0.883 (0.031) | 0.895 (0.029) | 0.130 (0.019) | 81.5 (3.0) | 0.83 (0.06) | 0.80 (0.06) | 0.81 (0.03) |
| LightGBM | 0.886 (0.031) | 0.891 (0.034) | 0.130 (0.019) | 81.4 (2.3) | 0.81 (0.04) | 0.82 (0.08) | 0.81 (0.03) |
| MLP | 0.903 (0.029) | 0.913 (0.024) | 0.117 (0.020) | 83.4 (3.3) | 0.88 (0.03) | 0.77 (0.10) | 0.82 (0.05) |
|  |  |  |  |  |  |  |  |
| **3-month mortality** | | | | | | | |
| LogReg | 0.824 (0.118) | 0.606 (0.197) | 0.076 (0.023) | 90.1 (2.6) | 0.61 (0.41) | 0.29 (0.24) | 0.37 (0.28) |
| LightGBM | 0.845 (0.110) | 0.623 (0.175) | 0.073 (0.023) | 90.6 (2.7) | 0.68 (0.36) | 0.38 (0.29) | 0.44 (0.29) |
| MLP | 0.878 (0.083) | 0.661 (0.183) | 0.067 (0.022) | 91.2 (3.0) | 0.79 (0.27) | 0.39 (0.27) | 0.48 (0.28) |
| The values are aggregated in all folds and expressed as mean (SD).  Abbreviations: AUROC, area under the receiver operating characteristic curve; AUPRC, area under the precision-recall curve; ACC, accuracy; LightGBM, light gradient-boosting machine; LogReg, logistic regression; MLP, multi-layer perceptron. | | | | | | | |

| **Table S4. Detailed performance and net reclassification improvement at low levels of false positive rate for prediction of 3-month unfavorable functional outcome** | | | | | | | |
| --- | --- | --- | --- | --- | --- | --- | --- |
|  | **FPR** | **Model** | **Sensitivity** | **PPV** | **NPV** | **NRI** | **Diagnostic OR** |
| **Internal validation dataset** | | | | | | | |
|  | 5% | LogReg | 0.507 [0.437 – 0.578] | 0.909 [0.889 – 0.928] | 0.670 [0.630 – 0.710] | Reference | 20.599 [14.155 – 27.043] |
|  |  | LGBM | 0.532 [0.443 – 0.620] | 0.913 [0.893 – 0.932] | 0.681 [0.634 – 0.729] | 0.023 [-0.063 – 0.109] | 22.977 [14.308 – 31.645] |
|  |  | MLP | 0.577 [0.497 – 0.656] | 0.919 [0.902 – 0.936] | 0.703 [0.661 – 0.745] | 0.066 [-0.003 – 0.135] | 27.415 [18.715 – 36.115] |
|  | 10% | LogReg | 0.652 [0.572 – 0.733] | 0.864 [0.843 – 0.885] | 0.731 [0.681 – 0.780] | Reference | 17.713 [11.221 – 24.204] |
|  |  | LGBM | 0.662 [0.595 – 0.728] | 0.867 [0.849 – 0.886] | 0.736 [0.694 – 0.778] | 0.010 [-0.052 – 0.073] | 18.577 [12.917 – 24.237] |
|  |  | MLP | 0.679 [0.615 – 0.743] | 0.871 [0.852 – 0.890] | 0.747 [0.703 – 0.790] | 0.028 [-0.019 – 0.074] | 20.227 [13.728 – 26.726] |
|  | 20% | LogReg | 0.783 [0.729 – 0.837] | 0.796 [0.774 – 0.817] | 0.792 [0.743 – 0.841] | Reference | 15.175 [10.314 – 20.036] |
|  |  | LGBM | 0.800 [0.756 – 0.844] | 0.799 [0.778 – 0.819] | 0.805 [0.763 – 0.848] | 0.017 [-0.023 – 0.057] | 16.700 [12.110 – 21.290] |
|  |  | MLP | 0.805 [0.755 – 0.855] | 0.799 [0.778 – 0.821] | 0.809 [0.763 – 0.855] | 0.022 [-0.014 – 0.058] | 17.261 [11.634 – 22.888] |
| **External validation dataset** | | | | | | | |
|  | 5% | LogReg | 0.430 [0.351 – 0.510] | 0.905 [0.879 – 0.931] | 0.612 [0.565 – 0.660] | Reference | 15.463 [9.953 – 20.973] |
|  |  | LGBM | 0.532 [0.439 – 0.626] | 0.922 [0.898 – 0.946] | 0.658 [0.601 – 0.716] | 0.109 [0.024 – 0.195] | 23.674 [13.531 – 33.817] |
|  |  | MLP | 0.499 [0.426 – 0.572] | 0.918 [0.899 – 0.937] | 0.642 [0.595 – 0.690] | 0.074 [0.018 – 0.130] | 20.632 [14.055 – 27.208] |
|  | 10% | LogReg | 0.560 [0.473 – 0.647] | 0.858 [0.832 – 0.884] | 0.663 [0.609 – 0.717] | Reference | 12.169 [7.887 – 16.450] |
|  |  | LGBM | 0.678 [0.603 – 0.754] | 0.879 [0.857 – 0.901] | 0.729 [0.675 – 0.784] | 0.123 [0.018 – 0.228] | 20.079 [12.789 – 27.369] |
|  |  | MLP | 0.625 [0.545 – 0.706] | 0.870 [0.847 – 0.894] | 0.698 [0.645 – 0.752] | 0.068 [0.008 – 0.128] | 15.931 [9.872 – 21.990] |
|  | 20% | LogReg | 0.708 [0.646 – 0.770] | 0.792 [0.762 – 0.823] | 0.723 [0.675 – 0.772] | Reference | 10.160 [7.127 – 13.194] |
|  |  | LGBM | 0.779 [0.720 – 0.837] | 0.808 [0.779 – 0.836] | 0.775 [0.729 – 0.822] | 0.076 [0.007 – 0.144] | 14.820 [9.931 – 19.709] |
|  |  | MLP | 0.770 [0.713 – 0.827] | 0.805 [0.779 – 0.832] | 0.768 [0.720 – 0.817] | 0.066 [0.017 – 0.114] | 14.018 [9.608 – 18.427] |
| The values are expressed as mean [95% CI].  Abbreviations: FPR, false positive rate; LGBM, light gradient-boosting machine; LogReg, logistic regression; MLP, multi-layer perceptron; NPV, negative predictive value; NRI, net reclassification improvement; OR, odds ratio; PPV, positive predictive value. | | | | | | | |

| **Table S5. Detailed performance and net reclassification improvement at low levels of false positive rate for prediction of 3-month mortality** | | | | | | | |
| --- | --- | --- | --- | --- | --- | --- | --- |
|  | **FPR** | **Model** | **Sensitivity** | **PPV** | **NPV** | **NRI** | **Diagnostic OR** |
| **Internal validation dataset** | | | | | | | |
|  | 5% | LogReg | 0.251 [0.171 – 0.331] | 0.430 [0.344 – 0.517] | 0.899 [0.880 – 0.917] | Reference | 6.879 [4.051 – 9.708] |
|  |  | LGBM | 0.263 [0.180 – 0.347] | 0.440 [0.353 – 0.526] | 0.900 [0.881 – 0.920] | 0.011 [-0.072 – 0.094] | 7.293 [4.015 – 10.570] |
|  |  | MLP | 0.291 [0.188 – 0.393] | 0.464 [0.372 – 0.556] | 0.904 [0.884 – 0.924] | 0.036 [-0.027 – 0.100] | 8.402 [4.412 – 12.393] |
|  | 10% | LogReg | 0.388 [0.290 – 0.486] | 0.363 [0.284 – 0.442] | 0.913 [0.895 – 0.930] | Reference | 6.098 [3.563 – 8.633] |
|  |  | LGBM | 0.450 [0.332 – 0.567] | 0.395 [0.315 – 0.475] | 0.921 [0.901 – 0.940] | 0.071 [-0.056 – 0.197] | 7.834 [4.186 – 11.482] |
|  |  | MLP | 0.497 [0.395 – 0.599] | 0.419 [0.352 – 0.485] | 0.927 [0.908 – 0.945] | 0.125 [0.052 – 0.198] | 9.400 [5.507 – 13.293] |
|  | 20% | LogReg | 0.645 [0.554 – 0.735] | 0.318 [0.265 – 0.370] | 0.941 [0.924 – 0.958] | Reference | 7.686 [4.505 – 10.867] |
|  |  | LGBM | 0.722 [0.637 – 0.806] | 0.342 [0.291 – 0.393] | 0.953 [0.938 – 0.969] | 0.093 [-0.036 – 0.222] | 10.990 [6.291 – 15.690] |
|  |  | MLP | 0.726 [0.634 – 0.818] | 0.343 [0.290 – 0.396] | 0.954 [0.938 – 0.970] | 0.099 [0.002 – 0.196] | 11.314 [5.995 – 16.633] |
| **External validation dataset** | | | | | | | |
|  | 5% | LogReg | 0.240 [0.113 – 0.367] | 0.307 [0.171 – 0.443] | 0.936 [0.917 – 0.955] | Reference | 6.808 [2.070 – 11.546] |
|  |  | LGBM | 0.325 [0.189 – 0.460] | 0.374 [0.242 – 0.507] | 0.942 [0.925 – 0.960] | 0.117 [-0.055 – 0.289] | 10.313 [4.051 – 16.574] |
|  |  | MLP | 0.267 [0.134 – 0.399] | 0.328 [0.193 – 0.463] | 0.938 [0.919 – 0.956] | 0.036 [-0.075 – 0.148] | 7.764 [2.283 – 13.246] |
|  | 10% | LogReg | 0.395 [0.260 – 0.530] | 0.262 [0.181 – 0.342] | 0.947 [0.928 – 0.965] | Reference | 6.582 [2.823 – 10.342] |
|  |  | LGBM | 0.470 [0.330 – 0.611] | 0.290 [0.215 – 0.366] | 0.953 [0.935 – 0.971] | 0.098 [-0.044 – 0.240] | 8.704 [3.632 – 13.775] |
|  |  | MLP | 0.421 [0.277 – 0.566] | 0.269 [0.180 – 0.359] | 0.949 [0.932 – 0.965] | 0.036 [-0.103 – 0.175] | 7.165 [2.872 – 11.457] |
|  | 20% | LogReg | 0.578 [0.443 – 0.713] | 0.206 [0.145 – 0.267] | 0.956 [0.940 – 0.973] | Reference | 5.921 [2.709 – 9.133] |
|  |  | LGBM | 0.651 [0.526 – 0.776] | 0.227 [0.164 – 0.290] | 0.964 [0.949 – 0.978] | 0.095 [-0.064 – 0.255] | 8.149 [3.517 – 12.782] |
|  |  | MLP | 0.595 [0.461 – 0.729] | 0.211 [0.150 – 0.272] | 0.958 [0.941 – 0.974] | 0.022 [-0.086 – 0.130] | 6.369 [2.809 – 9.930] |
| The values are expressed as mean [95% CI].  Abbreviations: FPR, false positive rate; LGBM, light gradient-boosting machine; LogReg, logistic regression; MLP, multi-layer perceptron; NPV, negative predictive value; NRI, net reclassification improvement; PPV, positive predictive value. | | | | | | | |

**Supplementary Figures**

**
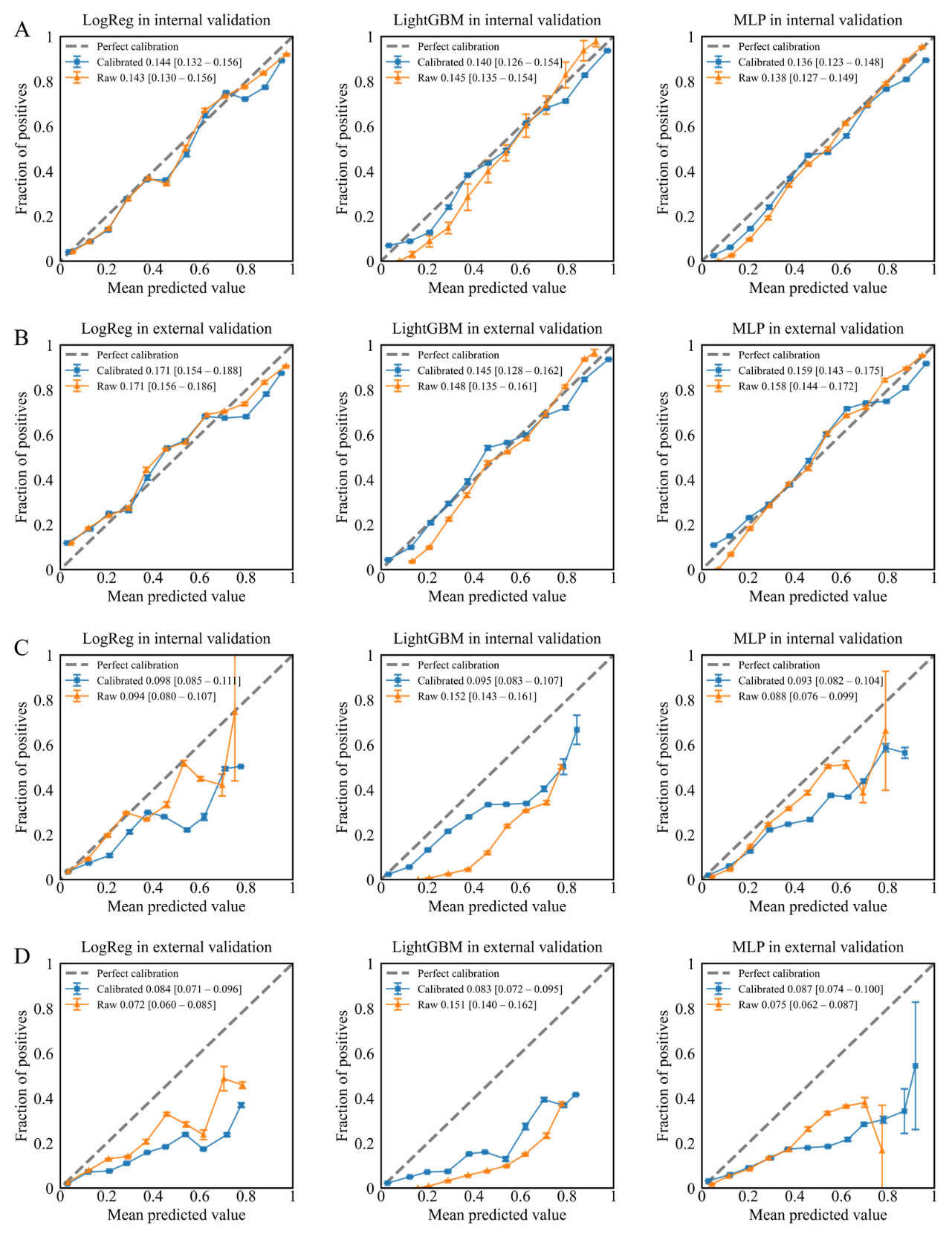
**

**Figure S1. Calibration curves before and after calibration of models.** Orange and blue lines represent calibration curves before (“Raw,” triangle markers) and after (“Calibrated,” cubic markers) calibration of the models, respectively. Error bars represent 95% confidence interval (CI) of the calibration curves, and the legend displays mean [95% CI] of the Brier scores for model prediction. **A, B**, Calibration curves for prediction of the 3-month unfavorable functional outcome. **C, D**, Calibration curves for the prediction of 3-month mortality. Abbreviations: LightGBM, light gradient-boosting machine; LogReg, logistic regression; MLP, multi-layer perceptron.

**
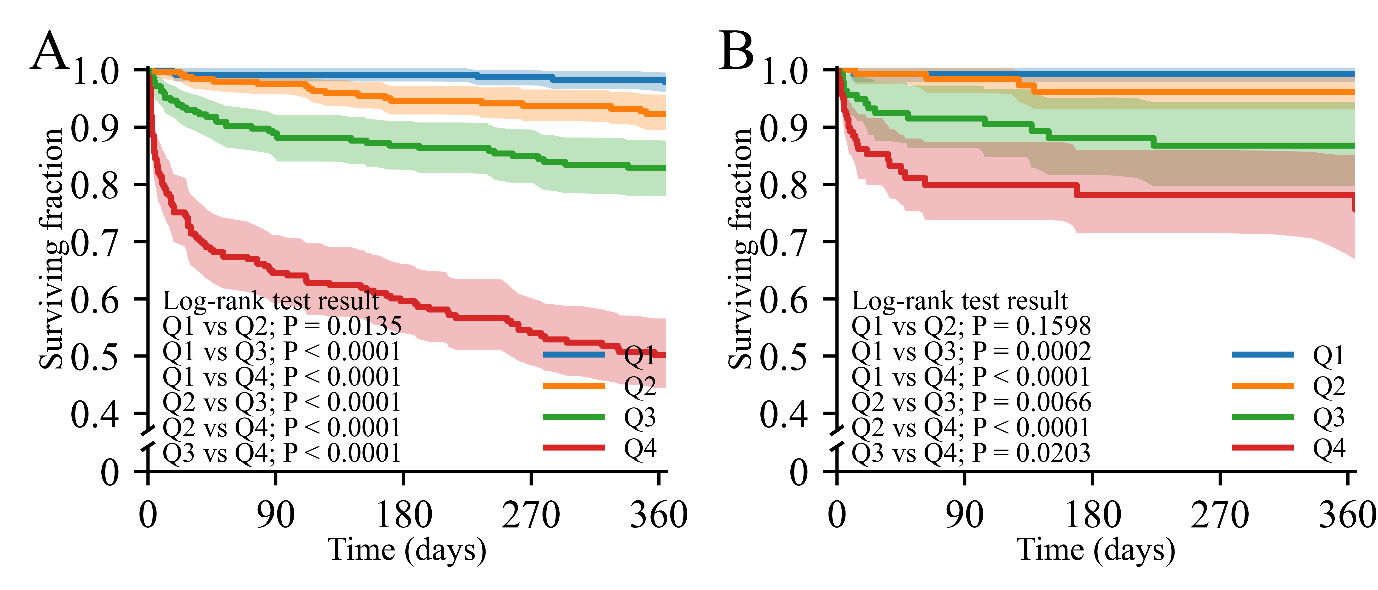
**

**Figure S2. Survival curves for four quartile strata obtained from the model for prediction of 3-month mortality.** MLP was used to obtain the four strata of internal validation data (A) and for those of external validation data (B). According to prediction score for 3-month mortality, Q1, Q2, Q3, and Q4 denote the first, second, third, and fourth quartile. Solid lines and shades represent mean curves and 95% confidence interval areas, respectively. Pair-wise comparisons between the curves were performed and *P* values are presented in the legend.

**
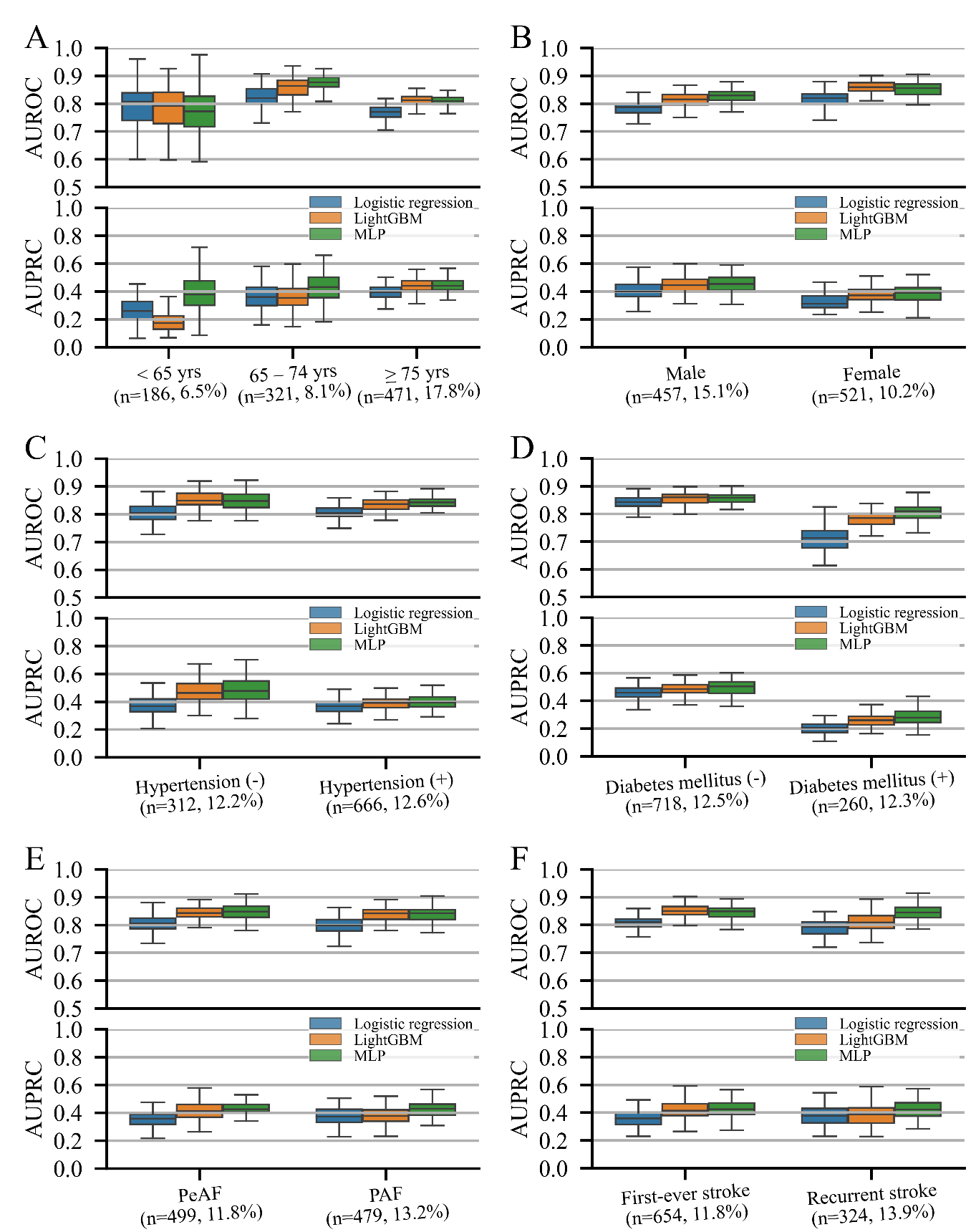
**

**Figure S3. Performance on different subgroup cohorts for prediction of 3-month mortality.** The area under the receiver operating characteristics curves and precision-recall curves. The notation (n = a, b%) under each name of a subgroup indicates the number of samples in the test set (a) and prevalence rate of the outcomes (b) of the subgroup. Box plots are plotted with whiskers of 1.5 times the interquartile ranges. Abbreviations: AUPRC, area under the precision-recall curve; AUROC, area under the receiver operating characteristics curve; DM, diabetic mellitus; HTN, hypertension; PAF, paroxysmal atrial fibrillation; PeAF, persistent atrial fibrillation.


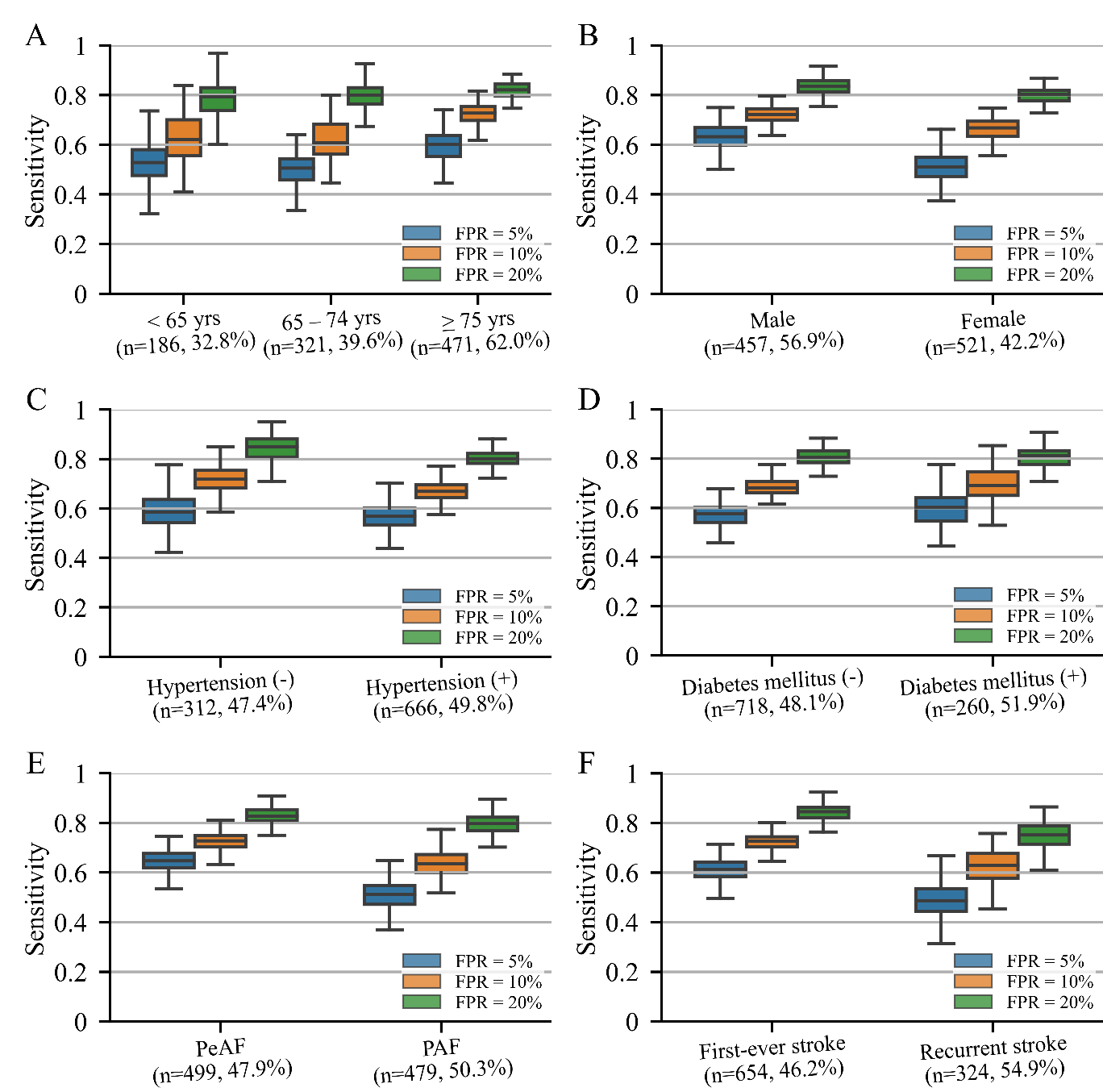


**Figure S4. Sensitivity at low levels of false positive rate on different subgroup cohorts for prediction the 3-month unfavorable functional outcome.** The trained multi-layer perceptron was used for the evaluation. The notation (n = a, b%) under each name of a subgroup indicates the number of samples in the test set (a) and prevalence rate of the outcomes (b) of the subgroup. Box plots are plotted with whiskers of 1.5 times the interquartile ranges. Abbreviations: FPR, false positive rate; HTN, hypertension; PAF, paroxysmal atrial fibrillation; PeAF, persistent atrial fibrillation; yrs, years.

**
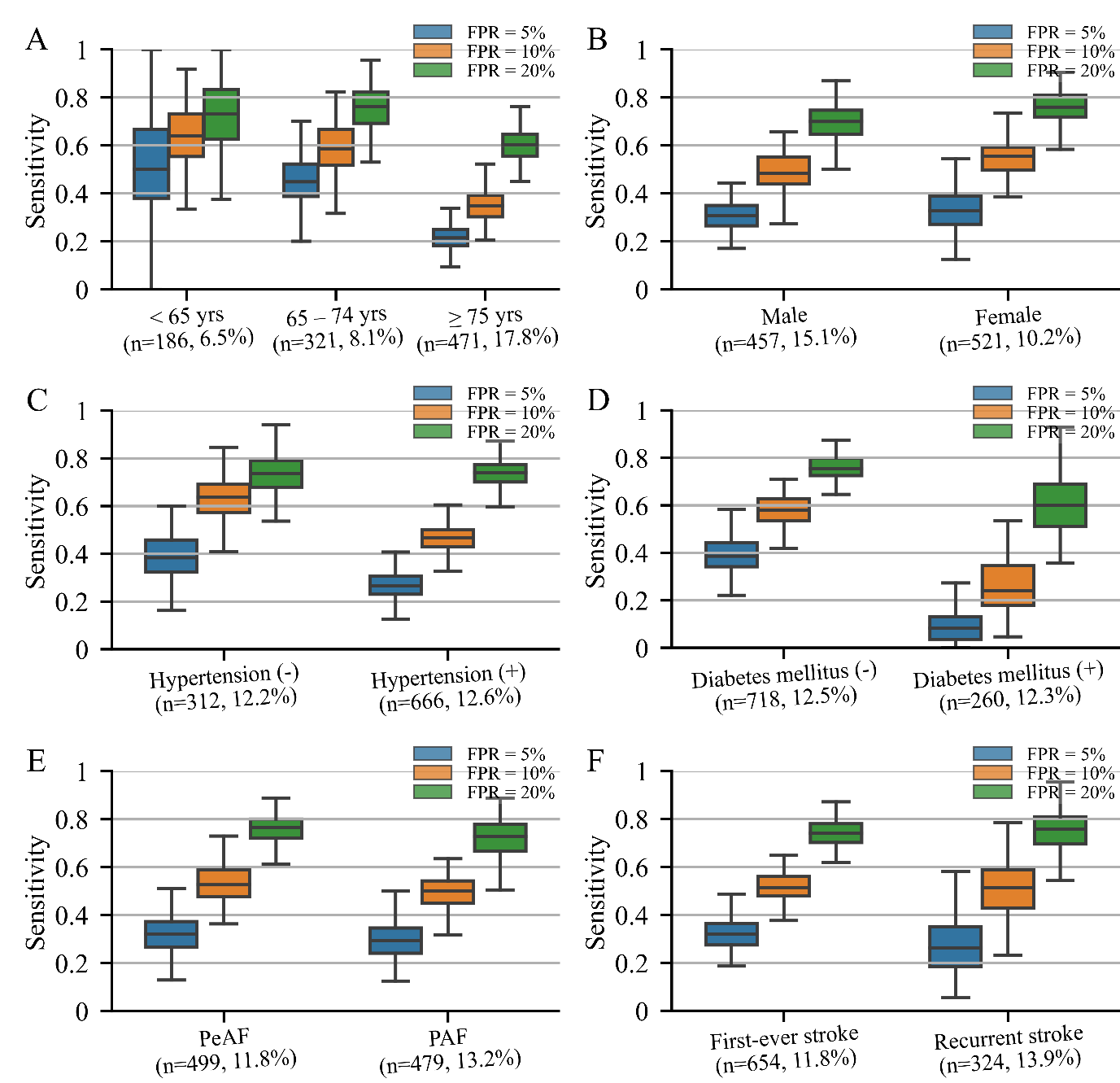
**

**Figure S5. Sensitivity at low levels of false positive rate on different subgroup cohorts for the prediction of 3-month mortality.** The trained multi-layer perceptron was used for the evaluation. The notation (n = a, b%) under each name of a subgroup indicates the number of samples in the test set (a) and prevalence rate of the outcomes (b) of the subgroup. Box plots are plotted with whiskers of 1.5 times the interquartile ranges. Abbreviations: FPR, false positive rate; HTN, hypertension; PAF, paroxysmal atrial fibrillation; PeAF, persistent atrial fibrillation; yrs, years.

**
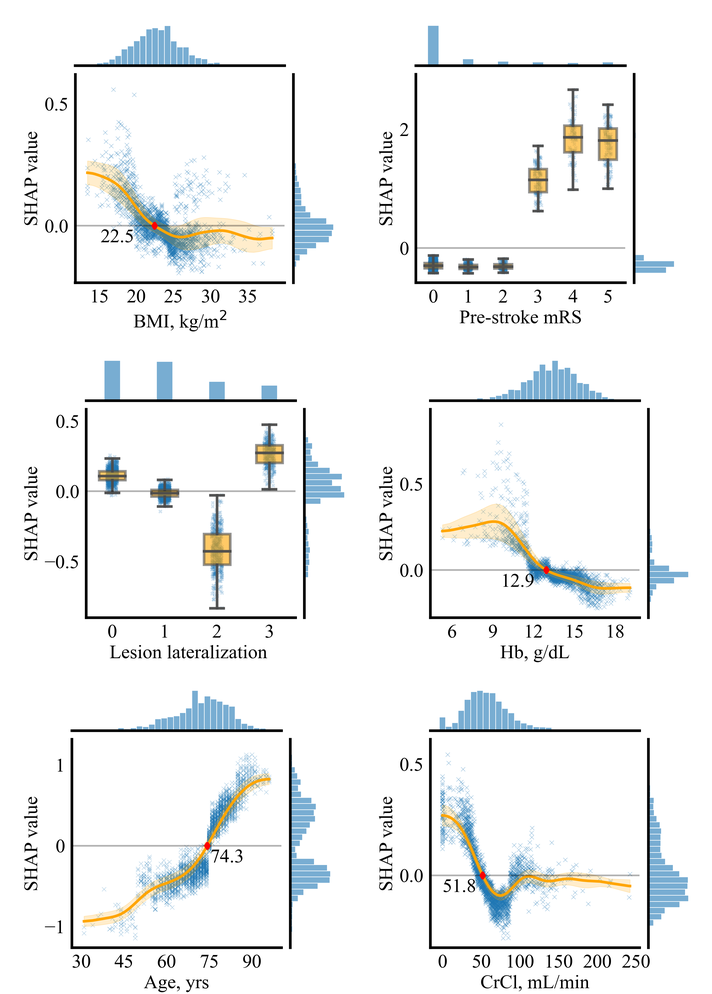
**

**Figure S6. Partial SHAP dependence plots for six representative variables for the prediction of the 3-month unfavorable functional outcome.** Histograms on the right and the top axes of each plot indicate distributions of SHAP and variable values, respectively. For continuous variables, scatter plots with regression lines represented with orange lines of mean and shades of SD are illustrated. Red diamonds represent cut-off values. For categorical variables, scatter plots with box plots with whiskers of 1.5 times the interquartile ranges are illustrated. The original labels of the numeric codes of *Lesion lateralization* variable are as follows: 0, Rt. anterior; 1, Lt. anterior; 2, Posterior; 3, Bilateral or diffuse multifocal.


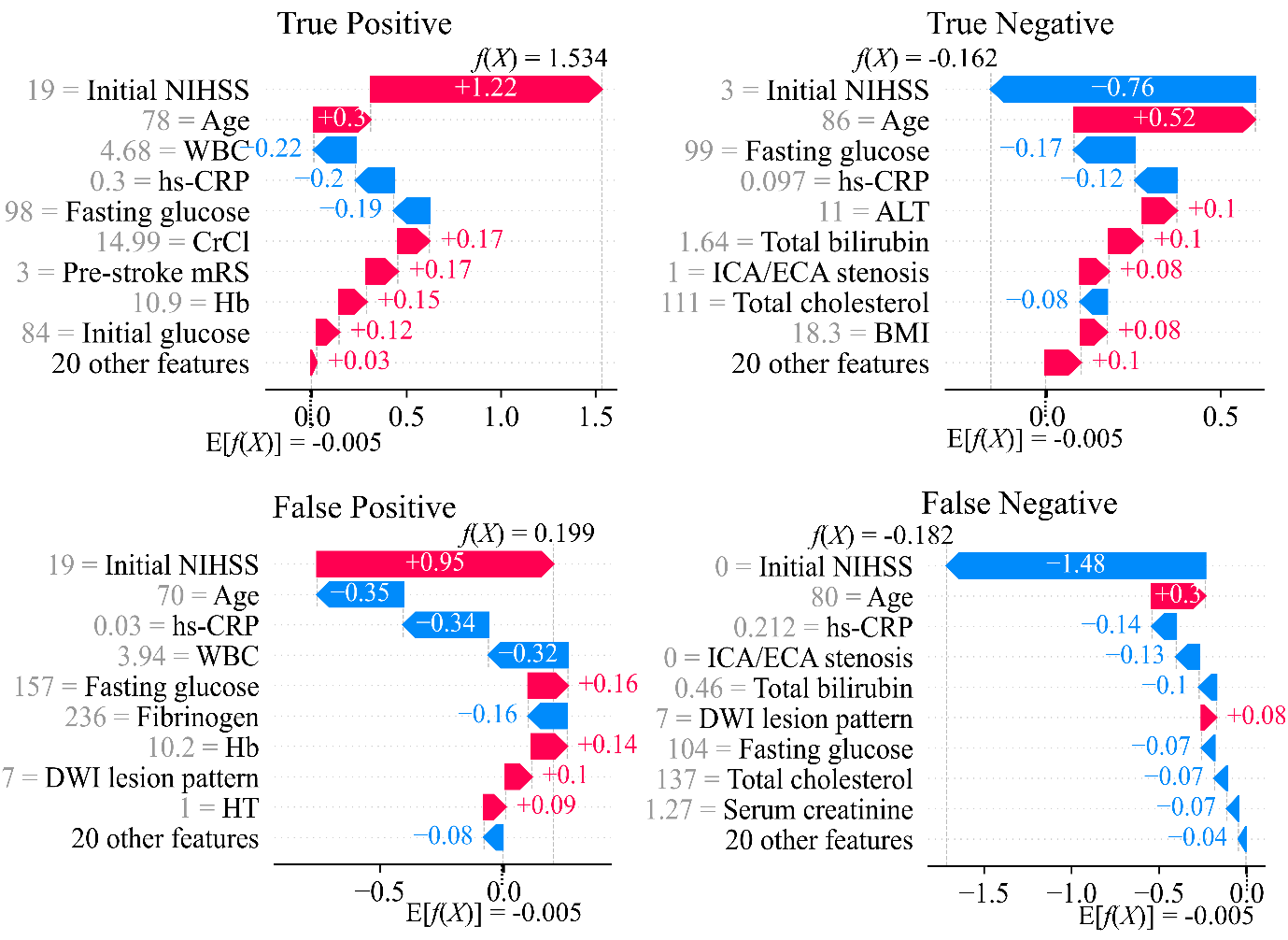


**Figure S7. Local interpretability of the developed gradient-boosted tree-based model for the prediction of 3-month unfavorable functional outcome.** E[*f*(*X*)] is a constant representing the base value, while *f*(*X*) is an output value representing the prediction score of the model. The cut-off threshold for the model’s decision was set at a false positive rate of 20%.

**
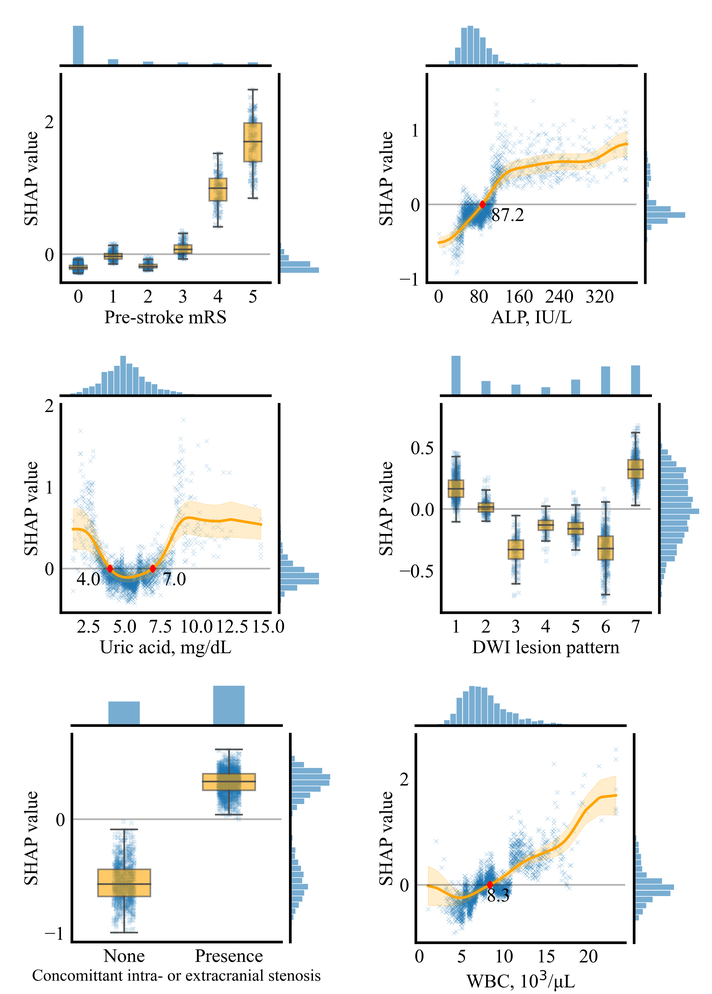
**

**Figure S8. Partial SHAP dependence plots for six representative variables for prediction of 3-month mortality.** Histograms on the right and the top axes of each plot indicate distributions of SHAP and variable values, respectively. For continuous variables, scatter plots with regression lines represented with orange lines of mean and shades of SD are illustrated. Red diamonds represent cut-off values. For categorical variables, scatter plots with box plots with whiskers of 1.5 times the interquartile ranges are illustrated. The original labels of the numeric codes of *DWI lesion pattern* variable are as follows: 1, single corticosubcortical; 2, cortical; 3, subcortical (≥ 15 mm); 4, subcortical (< 15 mm); 5, small scattered lesion in one vascular territory; 6, confluent and an additional lesion in one vascular territory; 7, multiple lesions in multiple vascular territories.


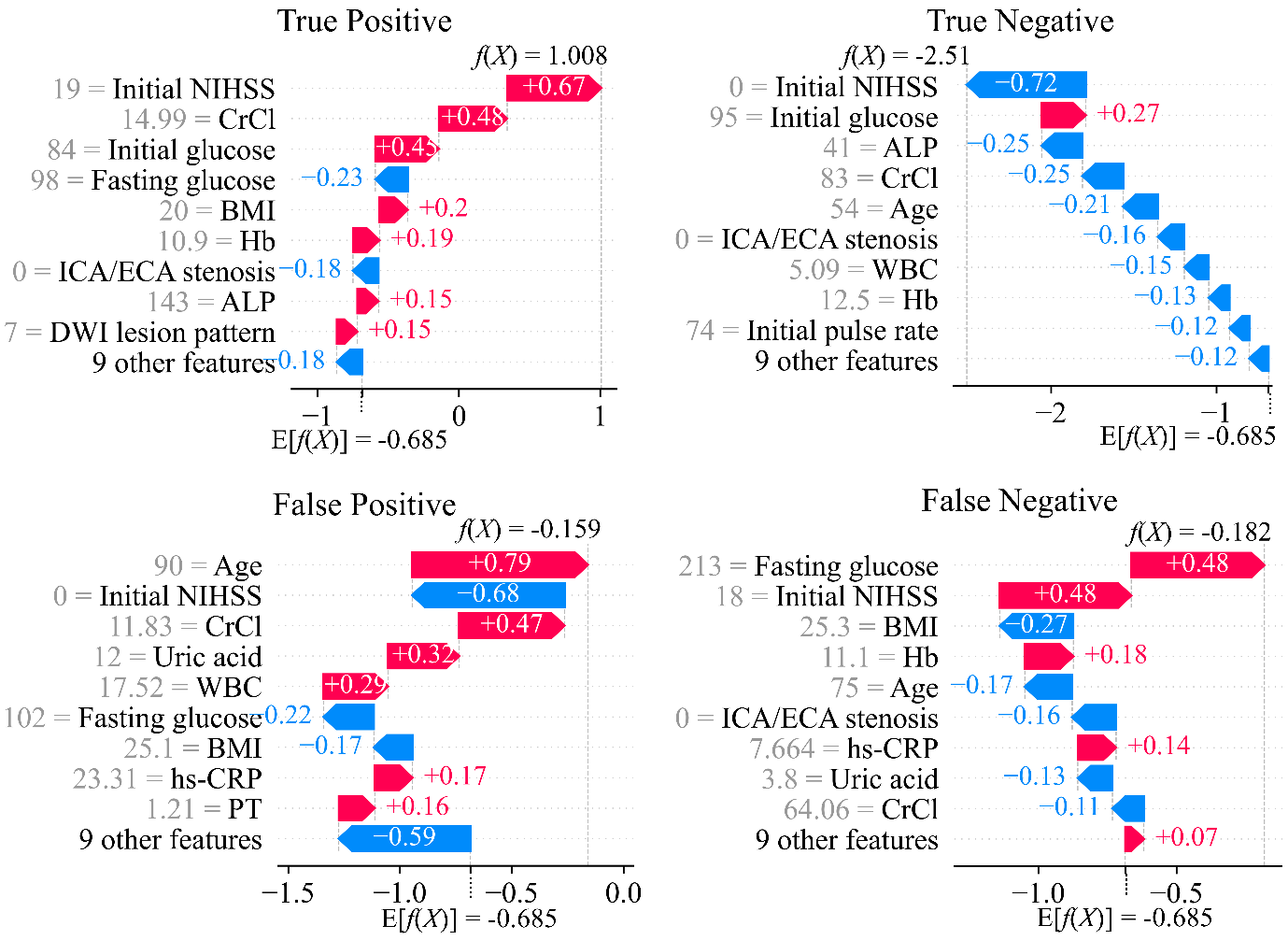


**Figure S9. Local interpretability of the developed gradient-boosted tree-based model for prediction of 3-month mortality.** E[*f*(*X*)] is a constant representing the base value, while *f*(*X*) is an output value representing the prediction score of the model. The cut-off threshold for the model’s decision was set at a false positive rate of 20%.
